# Rätselhafte B-Symptomatik bei einem 61-Jährigen unter Therapie einer rheumatoiden Arthritis

**DOI:** 10.1007/s00108-021-01090-1

**Published:** 2021-07-12

**Authors:** R. Wüstenberg, M. Christner, S. Schmiedel, A. Tariparast, D. Wichmann, M. Lennartz, H. Klose, S. Kluge

**Affiliations:** 1 II. Medizinische Klinik und Poliklinik, Abteilung für Pneumologie, Universitätsklinikum Hamburg-Eppendorf, Martinistr. 52, 20246 Hamburg, Deutschland; 2 I. Medizinische Klinik und Poliklinik, Sektion Infektiologie, Universitätsklinikum Hamburg-Eppendorf, Hamburg, Deutschland; 3 Institut für Medizinische Mikrobiologie, Virologie und Hygiene, Universitätsklinikum Hamburg-Eppendorf, Hamburg, Deutschland; 4 Klinik für Intensivmedizin, Universitätsklinikum Hamburg-Eppendorf, Hamburg, Deutschland; 5 Institut für Pathologie, Universitätsklinikum Hamburg-Eppendorf, Hamburg, Deutschland

**Keywords:** Histoplasmose, Granulome, Immunsuppression, Bronchoalveoläre Lavageflüssigkeit/Lymphozytose, Lungenkavernen, Histoplasmosis, Granuloma, Immunosuppression, Bronchoalveolar lavage fluid/lymphocytosis, Lung caverns

## Abstract

Ein Patient mit rheumatoider Arthritis sowie B‑Symptomatik, Polyneuropathie und einschmelzenden Lungenveränderungen unter Immunsuppression entwickelte nach zunächst subakutem Verlauf rasch progrediente zentrale neurologische Symptome und ein letales Multiorganversagen. Als ursächlich erwies sich eine disseminierte Infektion mit *Histoplasma capsulatum* unter Beteiligung des zentralen Nervensystems. Die Erstinfektion hatte sich fünf Jahre zuvor bei einem Karibikurlaub ereignet. Die Kombination aus Reiseaktivität und Immunsuppression erfordert die Berücksichtigung sonst in Deutschland sehr seltener infektiologischer Diagnosen.

## Abstract (en)

A patient with rheumatoid arthritis and immunosuppression developed symptoms of wasting, neuropathy and lung cavitations eventually leading to central nervous system symptoms and fatal multi-organ failure. Disseminated infection with *Histoplasma capsulatum* proved to be the underlying cause. The primary infection had apparently been acquired 4 years earlier on a holiday to the Caribbean. Rare infectious diseases should be considered in patients under immunosuppression and travel activities to specific endemic areas.

Aufgrund der globalen Reiseaktivität müssen im Einzelfall auch hierzulande seltene Infektionskrankheiten unter immunmodulatorischer Therapie berücksichtigt werden.

## Anamnese

Bei einem 61-jährigen, männlichen Patienten wurde im Februar 2014 eine seropositive rheumatoide Arthritis (RA) diagnostiziert. Die Behandlung erfolgte zunächst mit Methotrexat (MTX) bis maximal 25 mg s.c. wöchentlich und später als Kombination mit Leflunomid bis maximal 20 mg/Tag (Tab. 1).

**Table 1:** 

Methotrexat	2/2014–9/2018 (trockener Husten)
Leflunomid	5/2015–10/2018 und 2–5/2019 (Polyneuropathie)
Tofacitinib 10 mg/Tag	6–7/2019 (verschlechterter Allgemeinzustand)
Prednisolon 50 mg/Tag	Ab 7/2019

Ab Mitte 2018 beklagte der Patient Gewichtsverlust, Erschöpfungszustände, Schüttelfrost, Nachtschweiß, Arthralgien und Parästhesien der Füße (Tab. 2). Bei trockenem Husten wurde die MTX-Therapie wegen des Verdachts auf eine medikamentös-toxische Nebenwirkung beendet. Computertomographisch zeigten sich im Oktober 2018 multiple pulmonale Einschmelzungen mit Verdacht auf Abszess. In der bronchoalveolären Lavage (BAL) wurden *Staphylococcus aureus* und *Serratia marcescens* nachgewiesen und nach Resistogramm mit Moxifloxacin 400 mg/Tag über 6 Wochen behandelt. Die weiteren Kulturen (anaerob, Sprosspilze [Hefen], Schimmelpilze und Tuberkulose) ergaben keinen zusätzlichen Erkenntnisgewinn.

**Table 2:** 

Zeit	Maßnahmen und Befunde
2/2014	Erstdiagnose rheumatoide Arthritis, Rheumafaktor positiv, zyklische citrullinierte Peptide positiv
	*Therapie mit Methotrexat (maximal 25* *mg wöchentlich) seit 2/2014*
	*Therapie mit Leflunomid (maximal 20* *mg/Tag) seit 5/2015*
8–9/2018	Fatigue, Schüttelfrost, Nachtschweiß, Arthralgien, trockener Husten
	*Methotrexat abgesetzt (Therapiezeitraum 2/2014 bis 9/2018)*
10/2018	Einschmelzende Kavernen beidseits pulmonal. Histologisch: granulomatöse Entzündung
	8 Wochen Moxifloxacin bei Verdacht auf abszedierende Pneumonie (*Staphylococcus aureus, Serratia marcescens*)
	Trockener Husten persistiert
	*Leflunomid abgesetzt (Therapiezeitraum 5/2015 bis 10/2018)*
1/2019	Thorax-CT: rückläufige pulmonale Herde
2/2019	*Wiederbeginn Leflunomid*
4/2019	Thorax-CT: einschmelzende pulmonale Herde
	Progredienz der Lungenveränderungen, Progress der rheumatoiden Arthritis
	Transbronchiale Kryobiopsie: epitheloidzellige Granulome passend zu nekrotisierenden Rheumaknoten
5/2019	*Leflunomid abgesetzt (Therapiezeitraum 5/2015–10/2018 sowie 2–5/2019)*
6/2019	*Beginn Tofacitinib 10* *mg/Tag*
	Gewichtsverlust insgesamt 32 kg, Fatigue, Durstgefühl, Nykturie, trockener Husten
7/2019	Zusätzlich Schüttelfrost, Nachtschweiß, Fieber
	*Tofacitinib abgesetzt (Therapiezeitraum 6–7/2019)*
	Zusätzlich bulbäre Symptomatik, Halluzinationen, Mundsoor, Gedächtnislücken, Visusverschlechterung, Hörverlust
	*Beginn Prednisolon 50* *mg/Tag*
Ab 8/2019	Appetitlosigkeit, Gleichgewichtsstörungen, Zephalgien, Rücken‑/Nackenschmerzen, Einschränkung Feinmotorik, Verwirrtheit, Desorientiertheit
8/2019	Stationäre Aufnahme: cMRT (Abb. 3) ohne Hinweis auf entzündlichen Prozess
	Bronchoskopie: chronische Bronchitis, Kultur unauffällig
	Zentrumsverlegung zur weiteren Diagnostik und Therapie

*cMRT* kraniale Magnetresonanztomographie, *CT* Computertomographie

**Figure 1:**
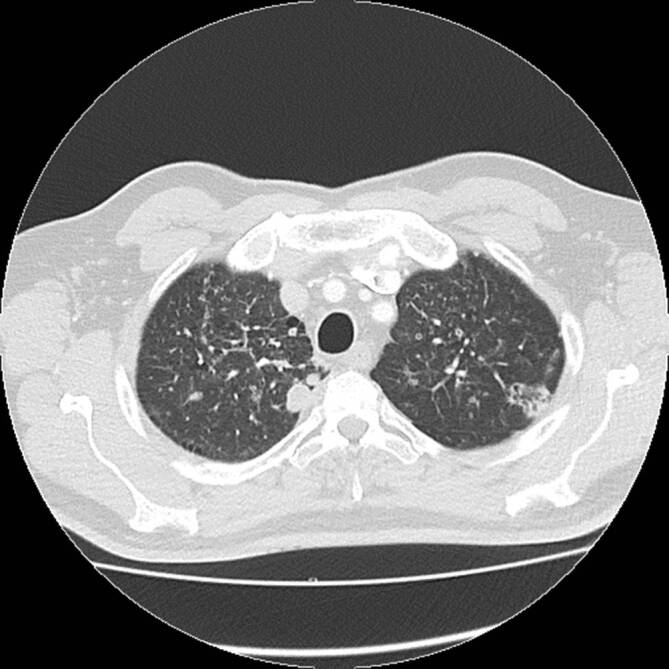

**Figure 2:**
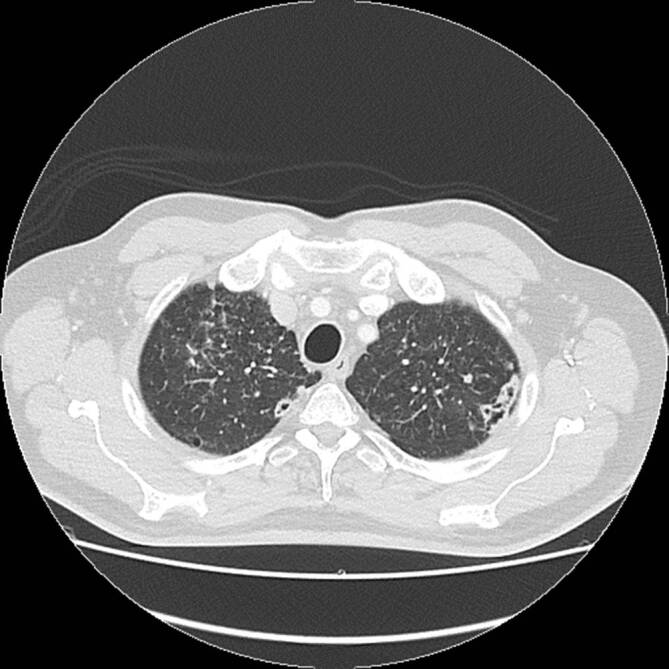

Die Leflunomidtherapie musste nach 4‑monatiger Pause bei zunehmender Aktivität der RA mit beidseitigen Knieschmerzen wieder aufgenommen werden. Die Lungenveränderungen waren erst regredient (Abb. 1), nun aber radiologisch wieder zunehmend (Abb. 2).

Aufgrund der Regredienz unter Antibiotikatherapie bei gleichzeitig nicht kontrollierter Rheumaaktivität wurde eine rheumatische Genese angezweifelt. Die Computertomographie (CT) des Thorax im April 2019 zeigte nunmehr einschmelzende Herde, zu deren pathogenetischer Einordnung vor Wiederaufnahme der Immunsuppression eine transbronchiale Kryobiopsie erfolgte. Diese ergab epitheloidzellige Granulome, die differenzialdiagnostisch nach direkter Mikroskopie sowie Hämatoxylin-Eosin-Färbung, Elastika-van-Gieson-Spezialfärbung, Grocott-Färbung, Ziehl-Neelsen-Färbung und Periodic-acid-Schiff-Reaktion als pulmonale Beteiligung der RA gewertet wurden. Die BAL zeigte ein mäßig granulozytäres und lymphozytäres Zellbild. Kulturell wurde kein Keim nachgewiesen. Ein interdisziplinäres Interstitial-lung-disease(ILD)-Board favorisierte nun nekrotisierende Rheumaknoten. Da keine infektiöse Ursache nachgewiesen werden konnte, wurde eine Intensivierung der Immunsuppression empfohlen.

Bei Arthralgien der Knie und vermuteter Polyneuropathie unter Leflunomid wurde die Therapie ab Juni 2019 auf Tofacitinib 10 mg/Tag umgestellt. In der Folge verschlechterte sich der körperliche Zustand des Patienten mit Fatigue, Polyurie, trockenem Husten, Nachtschweiß, Fieber und diabetischer Stoffwechsellage. Auch hier wurde eine medikamentös-toxische Genese diskutiert. Doch nach Umstellung von Tofacitinib auf Prednisolon 50 mg/Tag ab Mitte Juli 2019 persistierte das klinische Bild. Zusätzlich traten bulbäre Symptome, Verwirrtheit, Verschlechterung von Visus und Hörvermögen sowie Rücken- und Nackenschmerzen auf. Eine erweiterte neurologische Abklärung inklusive Elektroneuro- bzw. Elektromyographie, Autoantikörpern und Immunglobulinspiegeln deutete auf eine akute gemischte Polyneuropathie.

**Figure 3:**
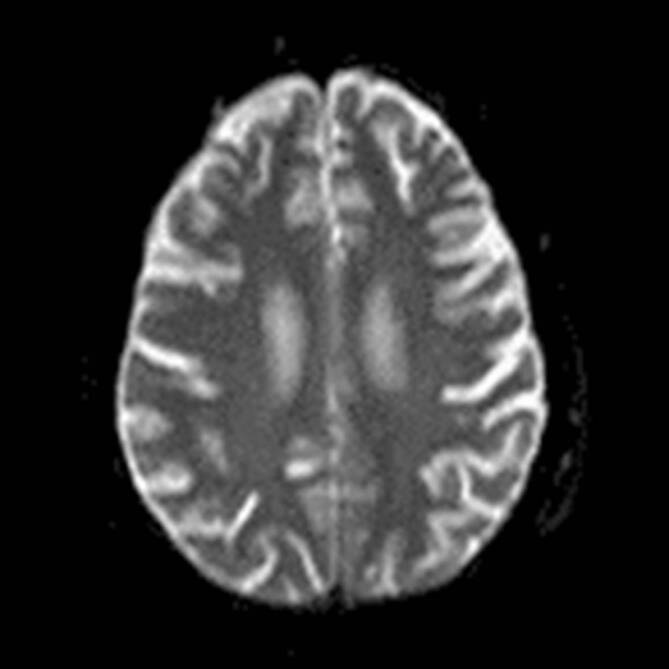

In einer kranialen Magnetresonanztomographie fanden sich keine Hinweise auf zerebrale Läsionen (Abb. 3).

Unter kalkulierter Therapie mit Ceftriaxon erfolgte im August 2019 die Verlegung in unser Zentrum bei unklarem entzündlichem und fraglich infektiösem Prozess unter Immunsuppression mit seit über einem Jahr bestehendem Beschwerdebild.

## Körperliche Untersuchung

Es präsentierte sich ein somnolenter Patient, nur zur Person orientiert, dysarthrisch und paraplegisch mit Inkontinenz. Die Sensibilität war seitengleich erhalten, Patella‑/Achillessehnenreflexe waren ausgefallen. Pyramidenbahnzeichen lagen nicht vor. Der Patient hatte rechts thorakale Schmerzen. Er bekam 4 l/min O_2_ über eine Nasenbrille. Ansonsten waren der kardiopulmonale Befund sowie Haut- und Schleimhäute unauffällig. Die Körpertemperatur betrug maximal 39,4 °C.

## Diagnostik

### Magnetresonanztomographie

Die *Kontrastmittelaufnahme der Cauda equina* ohne Beteiligung der kranialen Abschnitte passte zu einer Polyradikulitis oder einem Guillain-Barré-Syndrom.

### Computertomographie des Thorax

**Figure 4:**
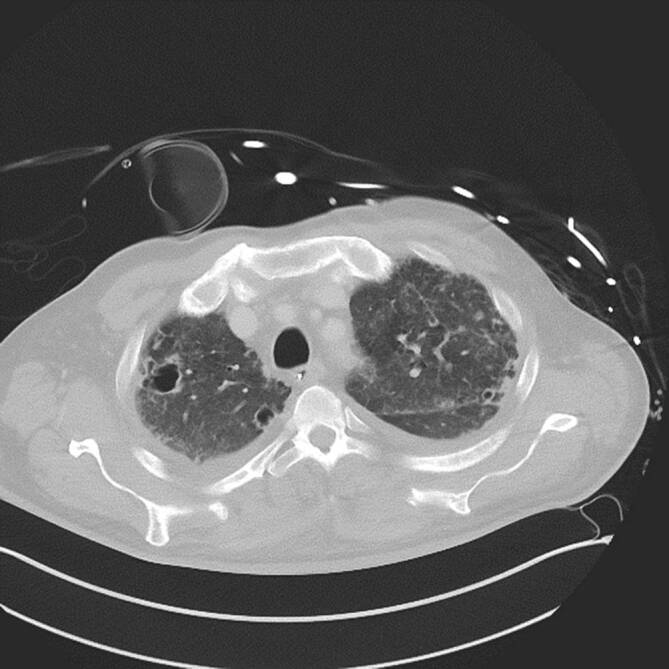

In der Computertomographie zeigten sich ubiquitär kavernisierende Läsionen mit Bronchusanschluss und Einschmelzungen, retikulogranulomatöse Veränderungen und eine progrediente Lymphadenopathie (Abb. 4 und 5).

**Figure 5:**
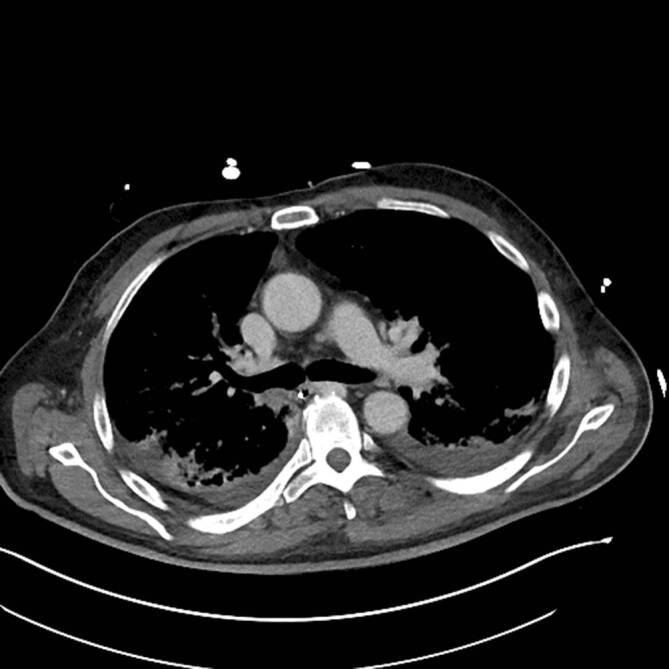

### Laboruntersuchung

Folgende Laborwerte wurden erhoben: Transaminasenerhöhung, Thrombopenie, Leukozyten 4,8 Mrd./l (Referenzbereich 3,8–11,0 Mrd./l), Neutrophile 3,88 Mrd./l (Ref. 1,50–7,70 Mrd./l) bzw. 80,6 % (kein Referenzbereich), Lymphozyten 0,62 Mrd./l (Ref. 1,1–3,4 Mrd./l) bzw. 12,8 % (kein Referenzbereich) im Differenzialblutbild, C‑reaktives Protein 79 mg/l (Ref. <5 mg/l), Prokalzitonin 0,88 µg/l (Ref. <0,5 µg/l).

## Therapie und Verlauf (Tab. 3)

Die *Liquorpunktion* ergab eine Leukozytose mit 1110/3 Zellen und eine erniedrigte Glukose <200 mg/l (Ref. 320–820 mg/l). Bei Verdacht auf Meningitis unter Immunsuppression wurde die antiinfektive Therapie auf Vancomycin, Meropenem, Ciprofloxacin und Aciclovir umgestellt sowie eine empirische antimykotische Therapie mit Caspofungin eingeleitet. Die *BAL* zeigte einen Lymphozytenanteil von 71 %.

**Table 3:** 

*Unklare Ausgangskonstellation*
– Rheumatoide Arthritis mit pulmonaler Beteiligung
– Unklare Pathogenese kavernös-einschmelzender Lungenherde und feinknotig-interstitielle Lungenerkrankung mit Tree-in-bud-Muster
– Progrediente diffuse neurologische Symptomatik
– C-reaktives Protein 70 mg/l, Leukozyten 4,6 Mrd./l, Prokalzitonin 0,88 μg/l unter immunsuppressiver Therapie
*Innerhalb von 36* *h Übernahme auf die Intensivstation bei Stupor, Thrombopenie und septischem Krankheitsbild*
– Verdacht auf Guillain-Barré-Syndrom
– Kalkulierte antimykotische Therapie mit Caspofungin
– Septischer Schock, hypoxische Insuffizienz, Intubation
*Xanthochromer Liquor*
– Pleozytose (granulozytär), erhöhtes Eiweiß (3514 mg/l, Ref. 140–500 mg/l), erniedrigte Glukose (<200 mg/l, Ref. 320–820 mg/l)
*Mikrobiologische Befunde*
– Mikroskopisch Sprosspilze (Hefen?), Kultur zunächst negativ
– *Candida*-PCR und *Cryptococcus*-PCR negativ
– β-D-Glukan-Test im Serum hoch positiv (483,5 pg/ml, Ref. < 11 pg/ml)
– Wechsel von Caspofungin auf liposomales Amphotericin B (5 mg/kgKG pro Tag i.v.)
– Nachweis von Histoplasma-DNA in Liquor, BAL
– Kultureller Nachweis von *Histoplasma capsulatum* aus Liquor, BAL und Blut
– Positives *Histoplasma*-Antigen im Urin
*Hyperakuter Verlauf trotz Maximaltherapie. Patient verstirbt im septischen Schock mit Multiorganversagen*

*BAL* bronchoalveoläre Lavage, *PCR* Polymerase-Kettenreaktion

Innerhalb von 36 h kam es zum septischen Multiorganversagen. Im Liquor wurden Sprosspilze nachgewiesen, die Therapie auf liposomales Amphotericin B 5 mg/kgKG pro Tag i.v. umgestellt. Bei zunächst negativer Pilzkultur, aber hohem β‑D-Glukan im Serum wurde erstmalig eine endemische Systemmykose vermutet. Mittels 18S-rRNA-Polymerase-Kettenreaktion (PCR) wurde letztlich *Histoplasma-capsulatum*-DNA im Liquor nachgewiesen. Durch Anzucht des Erregers aus Liquor, BAL-Flüssigkeit und Blut, Nachweis von *Histoplasma*-DNA in Liquor und BAL-Flüssigkeit sowie *Histoplasma*-Antigen im Urin wurde eine disseminierte Histoplasmose gesichert. Die Reiseanamnese ergab eine wahrscheinliche Erstinfektion bei einem Aufenthalt auf Curaçao 5 Jahre zuvor.

**Figure 6:**
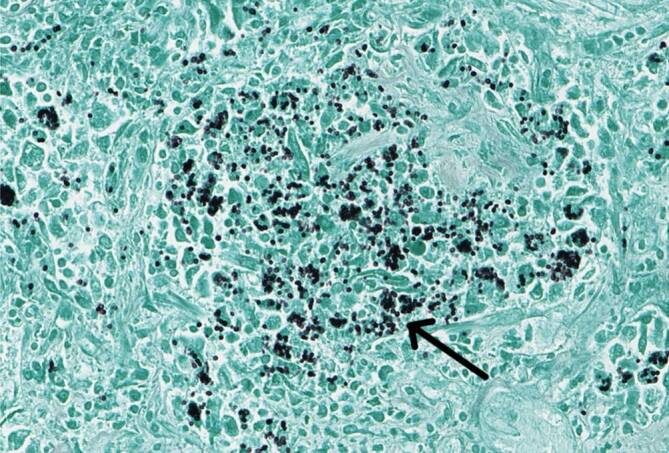

Trotz intensivmedizinischer Therapie verstarb der Patient im septischen Schock mit Multiorganversagen. Die Obduktion bestätigte den disseminierten Befall unter anderem von Lunge (Abb. 6) und zentralem Nervensystem.

## Diagnose

- Disseminierte Infektion mit *Histoplasma capsulatum* unter Beteiligung von Lunge und zentralem Nervensystem

## Diskussion

### Erreger und Epidemiologie der Histoplasmose

Die Histoplasmose wird durch den dimorphen Pilz *Histoplasma capsulatum* verursacht, der regional endemisch in Nord- bis Südamerika, der Karibik, Asien, Afrika und Australien auftritt [6]. Europa gilt bislang nicht als Endemiegebiet [2].

Ein erhöhtes Infektionsrisiko wird durch Exposition gegenüber Kot von Vögeln und Fledermäusen, verrottendem organischem Material und Erdarbeiten beschrieben [2]. Gemessen an der Expositionswahrscheinlichkeit in Endemiegebieten ist die Inzidenz der manifesten Histoplasmose niedrig. Immunkompromittierte Patienten gelten als besonders gefährdet für disseminierte Infektionen [6]. Infektionen treten oft im Zusammenhang mit Reiseaktivitäten in Endemiegebiete auf [8]. Reaktivierungen mit einer Latenz von mehreren Jahren sind möglich.

Reaktivierungen einer Histoplasmose sind auch nach mehrjähriger Latenz möglich

*H. capsulatum *lebt unter Umweltbedingungen als saprophytischer Schimmelpilz. Die Mikrokonidien (Sporen) werden mit Luftströmungen kilometerweit transportiert, die Inokulation erfolgt inhalativ. Bei Körpertemperatur wandelt sich die Konidie zur Hefeform und provoziert im Körper eine epitheloidzellig-granulomatöse Entzündung [5, 7].

Eine Mensch-zu-Mensch-Übertragung wurde nicht beschrieben. In Deutschland besteht für die Histoplasmose keine Meldepflicht, sodass keine genaue Inzidenz bekannt ist [6]. Hierzulande ist die Erkrankung jedoch eine Rarität.

### Krankheitsverlauf – Einfluss einer Immunsuppression

Der Verlauf variiert von asymptomatischen bis subklinischen Formen, unter anderem mit Panzytopenie, Hepatosplenomegalie, erhöhten Transaminasen, kutanen, oropharyngealen oder gastrointestinalen Läsionen bis hin zu akuten Verläufen mit zusätzlich Fieber, Abgeschlagenheit und Gewichtsverlust. In Endemiegebieten sind Human-immunodeficiency-virus(HIV)-Infektionen mit einer erhöhten Histoplasmoseinzidenz assoziiert. Die Erkrankung gilt als Stadium C, das ein „acquired immunodeficiency syndrome“ (AIDS) definiert.

Während bei Immungesunden die Infektion meist asymptomatisch oder selbstlimitierend verläuft, kommt es bei Immunsupprimierten zu schweren disseminierten, oft tödlichen Verläufen.

MTX, Leflunomid [3] und Tofacitinib inhibieren die T‑Zell-vermittelte Immunität und unter anderem die Signalwege über Interleukin‑6 und Interferon‑γ (IFN-γ). Für die granulomatöse Abwehrreaktion des Körpers sind vor allem IFN‑γ und Tumor-Nekrose-Faktor‑α notwendig [5]. Die Kombination von Tofacitinib und Glukokortikoiden gilt als Risikofaktor für schwere systemische Infektionen [4].

### Diagnostik

Die pulmonale Histoplasmose kann radiologisch mit retikulogranulomatösen Veränderungen, kavernisierenden Läsionen, Kalzifizierungen und einer Lymphadenopathie die Abgrenzung von einer Lungentuberkulose, Sarkoidose, dem pulmonalen Befall einer rheumatologischen Grunderkrankung oder medikamentös-toxischen Veränderungen erschweren [9]. Die BAL kann ein lymphozytäres Zellbild zeigen.

Disseminierte Infektionen können alle Organsysteme betreffen. Hervorzuheben ist das zentrale Nervensystem, unter anderem mit Verwirrtheit, Dysarthrie, Meningitis oder Polyradikulitis. Die hyperakute Verlaufsform kann zum fulminanten Multiorganversagen führen [9].

Der kulturelle Nachweis gilt als Goldstandard, die Wachstumszeit beträgt 2–6 Wochen. Die Antikörperbildung benötigt 4–8 Wochen und ist auch danach nicht zuverlässig nachweisbar. Der Nachweis von *Histoplasma*-Antigen im Urin mittels Enzymimmunoassay (EIA; nur in Speziallaboren) gilt bei schweren disseminierten Infektionen als ausreichend zuverlässig [1]. Spezifische real-time quantative PCR (qPCR)-Tests können bei Verdacht die Diagnose einer Histoplasmose bestätigen. PCR-Suchtests wie die 18S-rRNA-PCR sind in kommerziell erhältlichen Primer-Zusammenstellungen wenig sensitiv. Gegebenenfalls kann der mikroskopische Nachweis von Hefen im Organbiopsat (histologische Untersuchung) die Diagnose ermöglichen oder ein β‑D-Glukan-Test die Nutzung einer PCR-Diagnostik rechtfertigen.

Die Sensitivität der mikrobiologischen Diagnostik einer Histoplasmose wird durch Nutzung unterschiedlicher Verfahren und Untersuchungsmaterialien (beispielsweise Urin, Serum, Gewebe, BAL-Flüssigkeit, Liquor) erhöht. Bei positivem Urin-Antigen-EIA kann dieser zur Verlaufsbeurteilung herangezogen werden [6].

### Therapie

Zur Therapie der Histoplasmose wird Itraconazol empfohlen, In-vitro-Daten und klinische Fallserien deuten auf eine Wirksamkeit von Posaconazol, Voriconazol und Isavuconazol hin. Nicht empfohlen werden Fluconazol und Echinocandine [6].

Bei schwerer Erkrankung wird für die Initialtherapie Amphotericin B für 1–2 Wochen empfohlen, gefolgt von Itraconazol für mindestens 6 Wochen, gegebenenfalls auch lebenslang, abhängig von der zugrunde liegenden Krankheitskonstellation [6].

### Resümee

Zusammenfassend ist die Histoplasmose außerhalb von Endemiegebieten noch eine Ausnahmeerscheinung. Sie muss jedoch vor dem Hintergrund der Reiseaktivität, langer Latenzzeiten zwischen Exposition und Erkrankung, der Ausdehnung endemischer Areale sowie der Verbreitung immunmodulierender Medikationen bei unklaren Krankheitskonstellationen mit granulomatöser Entzündung berücksichtigt werden.

## Fazit für die Praxis

- Unter Immunmodulation müssen auch in Deutschland seltene Infektionskrankheiten differenzialdiagnostisch bedacht werden.
- Reaktivierungen endemischer Infektionen sind auch mit mehrjähriger Latenz möglich.
- Spätestens bei Auftreten zentralnervöser Symptome sollte eine Liquorpunktion erwogen werden.
- Der Antigennachweis im Urin wird über das Referenzzentrum des Robert Koch-Instituts angeboten.
- Als seltene Erkrankung sollte eine Histoplasmose in spezialisierten infektiologischen Zentren behandelt werden.
